# COVID-19-Related manuscripts: lag from preprint to publication

**DOI:** 10.1186/s13104-022-06231-9

**Published:** 2022-11-05

**Authors:** Emily Drzymalla, Wei Yu, Muin J. Khoury, Marta Gwinn

**Affiliations:** 1 Office of Genomics and Precision Public Health, Office of Science, Centers for Disease Control and Prevention, Atlanta, GA United States of America; 2 Tanaq Support Services, Atlanta, GA United States of America

**Keywords:** Preprint, COVID-19, Journal publication, Publication time, Data availability

## Abstract

**Objective:**

Preprints have had a prominent role in the swift scientific response to COVID-19. Two years into the pandemic, we investigated how much preprints had contributed to timely data sharing by analyzing the lag time from preprint posting to journal publication.

**Results:**

To estimate the median number of days between the date a manuscript was posted as a preprint and the date of its publication in a scientific journal, we analyzed preprints posted from January 1, 2020, to December 31, 2021 in the NIH iSearch COVID-19 Portfolio database and performed a Kaplan-Meier (KM) survival analysis using a non-mixture parametric cure model. Of the 39,243 preprints in our analysis, 7712 (20%) were published in a journal, after a median lag of 178 days (95% CI: 175–181). Most of the published preprints were posted on the bioRxiv (29%) or medRxiv (65%) servers, which allow authors to choose a subject category when posting. Of the 20,698 preprints posted on these two servers, 7358 (36%) were published, including approximately half of those categorized as biochemistry, biophysics, and genomics, which became published articles within the study interval, compared with 29% categorized as epidemiology and 26% as bioinformatics.

**Supplementary Information:**

The online version contains supplementary material available at 10.1186/s13104-022-06231-9.

## Introduction

Preprints, which are research manuscripts posted online prior to peer-reviewed journal publication, have become increasingly popular in biomedical research during the last decade [1]. The use of preprints has advantages for authors, allowing them to share their work quickly with peers and the public without cost [2, 3]. Preprints also offer advantages to the scientific community, accelerating scientific communication by sharing study results before and during the peer-review process, which may take months [3].

The scientific response to the COVID-19 pandemic produced a surge of research publications, including more than 30,000 preprints by the end of 2020. Several studies have analyzed the characteristics and contributions of preprints related to COVID-19 [4–6]. For example, studies conducted early in the pandemic reported that 5.7% of COVID-19 preprints resulted in journal publications; these preprints were published more quickly and cited more often than non-COVID-19 preprints [4, 5]. Now, two years into the COVID-19 pandemic, we examined how the preprint literature has evolved with a particular focus on the time interval between preprint and journal publication.

## Main text

### Methods

Several specialized databases have been established to capture COVID-19 research findings [7–9]. In April 2020, the NIH Office of Portfolio Analysis launched the iSearch COVID-19 Portfolio as a comprehensive, curated database of COVID-19 publications from Pubmed [7] and preprints from eight preprint servers. Although the iSearch COVID-19 Portfolio database links some preprints with their subsequent journal publications, this linkage is incomplete. On January 20, 2022, we downloaded from the iSearch COVID-19 Portfolio all preprints with a publication date from January 1, 2020, to December 31, 2021, along with all available links from preprints to their subsequent journal publications. We developed an automatic script to scan PubMed for possible preprint-publication matches that iSearch COVID-19 Portfolio might have missed and retrieved the PubMed Epub date for each PubMed records using NCBI utilities [10].

When available, we used the PubMed Epub date as the journal publication date, since many journals publish accepted manuscripts online before they appear in print. For each preprint in the iSearch COVID-19 Portfolio, we calculated the preprint-to-publication date by subtracting the preprint date from the journal publication date. The iSearch COVID-19 Portfolio dataset contains only a date for the most recent version of each preprint; however, version information is available for preprints published on medRxiv and bioRxiv. For each preprint published on these servers, we retrieved the date when the first version was published along with other metadata using the bioRxiv/medRxiv API [11]. Some data cleaning was done before analysis (see detail in appendix). To estimate the median number of days from preprint to publication, we performed a Kaplan-Meier (KM) survival analysis using a non-mixture parametric cure model with the R package “flexsurvcure” (version 1.2.0) [12]. This model also allowed us to estimate the “cure fraction,” i.e., the proportion of preprints that would never be published in a scientific journal.

## Results

**Fig. 1:**
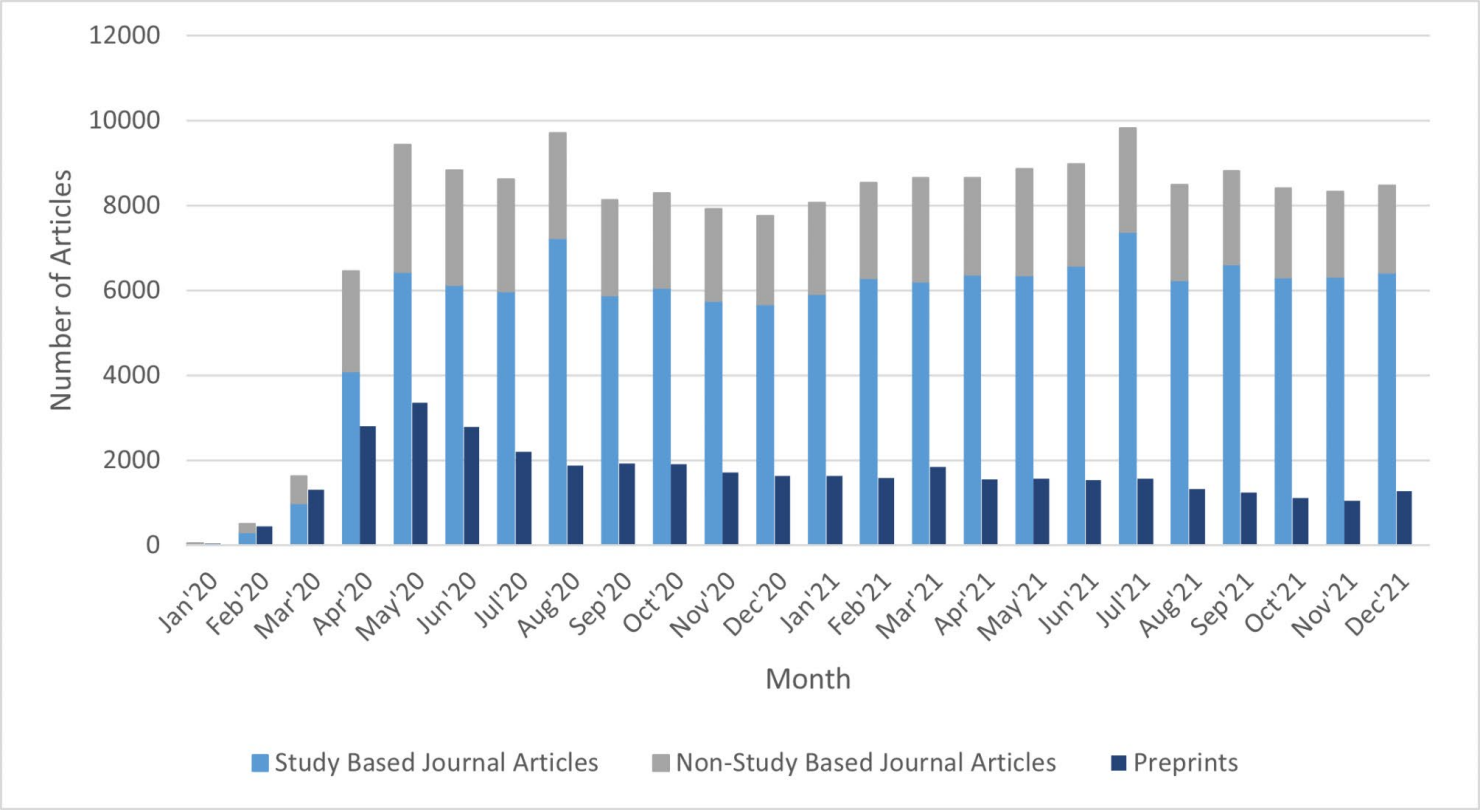
COVID-19-related preprints and journal articles published, by month, January 1, 2020 to December 31, 2021. (^a^ – study based journal articles refers to articles that analyzed data such as observational studies, trials, and meta-analyses. ^b^ – non-study based journal articles refers to articles that did not analyze data such as commentaries.)

The iSearch COVID-19 Portfolio database from January 1, 2020, to December 31, 2021, included 216,651 publications. Of these, 39,243 (20%) were preprints published on one of eight preprint servers: medRxiv (39%), Research Square (21%), SSRN (12%), bioRxiv (13%), arXiv (10%), Preprints.org (3.4%), ChemRxiv (1.5%), and Qeios (0.14%). The monthly number of new preprints peaked in May 2020 at 3453, plateauing after August 2020 at a level of approximately 1000–2000 new preprints per month. More journal articles than preprints were published each month, even in January 2020. Preprint platforms such as bioRxiv and medRxiv typically do not publish non-research articles like commentaries, news, or editorials [11–14]. When such articles are excluded, limiting published articles only to those reporting study results, preprints outnumbered journal publications only in January and February 2020 (Fig. 1).

We found a corresponding journal publication for 7712 (20%) of all preprints, including 7614 linked in the iSearch COVID-19 Portfolio dataset and 98 more by using our matching algorithm. The proportion of preprints that became journal publications varied among preprint servers: 65% of preprints on medRxiv, 29% on bioRxiv, 5% on SSRN, 0.7% on Research Square, 0.2% on arXiv, 0.1% on Preprints.org, 0.03% on chemRxiv, and 0% of preprints on Qeios.

**Fig. 2:**
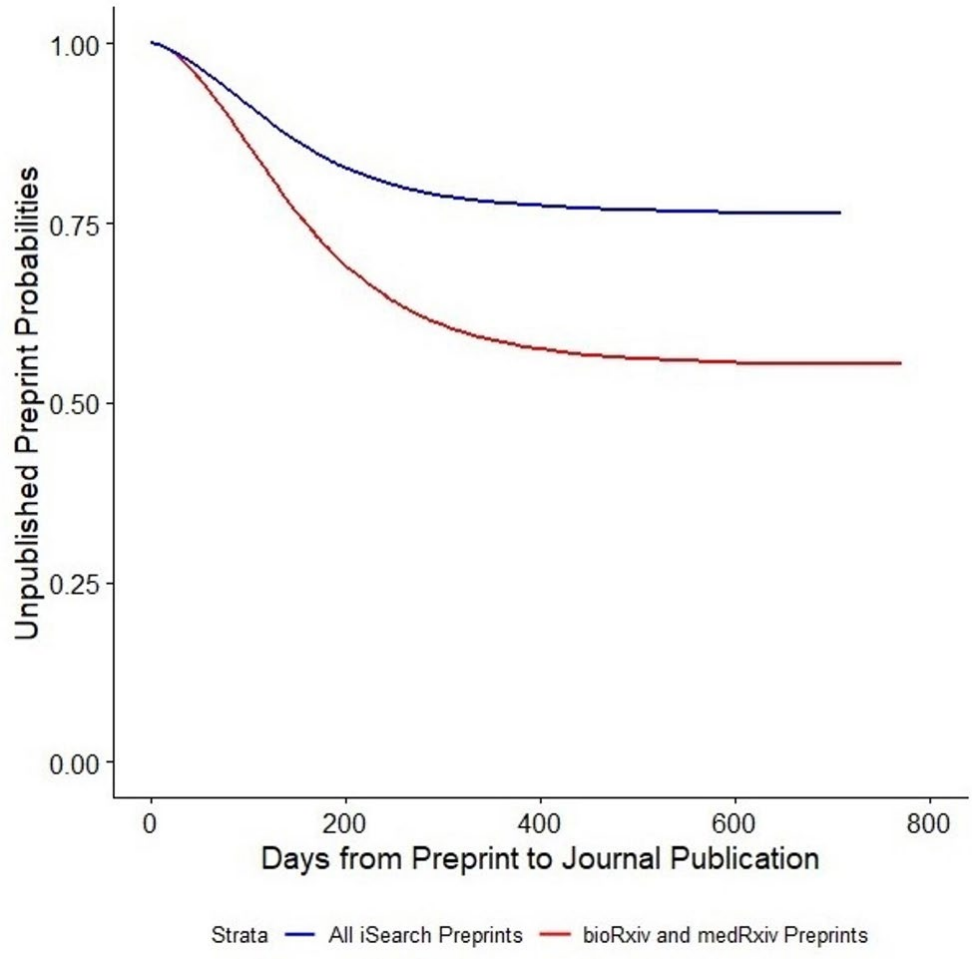
Survival curve for days from COVID-19-related preprint posting to journal publication

The interval from preprint posting to journal publication ranged from 1 to 614 days, with a median of 178 days (95% CI: 175–181) estimated by the Kaplan-Meier analysis (Fig. 2). Articles corresponding to the 7847 preprints were published in 1462 different journals. The journals publishing the largest numbers of these articles were PLoS One (n = 597), Scientific Reports (n = 265), and Nature Communications (n = 183), which together accounted for approximately 14%. The median number of days from preprint to publication in PLoS One was 196 (95% CI: 187–206), compared with 232 (95% CI: 218–246) in Scientific Reports and 214 (95% CI: 197–232) in Nature Communications. For all other journals, the median number of days from preprint to publication was 167 (95% CI: 163–170).

Of the 20,698 preprints posted on medRxiv or bioRxiv, 7358 (36%) had become journal publications by the time of our study. The number of versions of each preprint ranged from 1 to 11 but most (71%) existed as only a single version. The time from preprint to publication for the bioRxiv and medRxiv preprints ranged from 1 day to 615 days, with an estimated median of 205 days (95% CI: 201–209) (Fig. 2). From our survival analysis, the estimated cure fractions were 0.765 for all preprints in iSearch and 0.555 for the preprints in bioRxiv and medRxiv.

Authors submitting preprints to medRxiv and bioRxiv can choose to tag them with one of 76 subject areas; the leading categories are listed in Table 1. More than half of the preprints in the biochemistry, biophysics, and genomics categories became published articles, compared with 29% in epidemiology and 26% in bioinformatics.

**Table 1:** COVID-19-related preprints posted on bioRxiv and medRxiv by top 10 author-selected subject area

Top 10 Subject Area	Number of Published Preprints	Percent of All Published Preprints	Percent of Published Preprints for each Subject Area
infectious diseases	2216	30% (2216/7358)	36% (2216/6106)
epidemiology	1176	16% (1176/7358)	29% (1176/4028)
microbiology	684	9% (684/7358)	49% (684/1402)
public and global health	535	7% (535/7358)	31% (535/1712)
immunology	435	6% (435/7358)	44% (435/1000)
bioinformatics	229	3% (229/7358)	26% (229/584)
biochemistry	173	2% (173/7358)	53% (173/329)
biophysics	152	2% (152/7358)	53% (152/288)
genomics	149	2% (149/7358)	50% (149/300)
molecular biology	112	2% (112/7358)	41% (112/270)
Total	5861	80% (5861/7358)	37% (5861/16,019)

## Discussion

Two years into the COVID-19 pandemic, we found that 20% of all COVID-19-related preprints on the eight major preprint servers monitored by NIH’s iSearch COVID-19 Portfolio database had been published in scientific journals. We estimated that the median interval between preprint and publication was 178 days overall and only slightly longer (205 days) when calculated from the date of the first preprint version (available only from bioRxiv and medRxiv). Our findings contrast with those from analyses published early in the pandemic. For example, an analysis based on iSearch COVID-19 Portfolio data from January 1, 2020, to May 31, 2020, reported that only 5.7% of preprints had become journal publications, after a median interval of 110 days [5]. An analysis of all medRxiv preprints posted from January 1, 2020, to June 30, 2020, calculated that the median days from preprint to journal publication was 46 days for COVID-19 preprints, compared with 141 days for all other preprints [4]. An analysis of bioRxiv preprints posted before the pandemic began found a median of 166 days from preprint posting to journal publication [1]. Our estimated median of 178 days suggests that as the pandemic continues, the interval to journal publication for COVID-19 preprints is becoming more like that for non-COVID-19 preprints.

Because more recent preprints have had less time to become journal publications, estimating the time to publication based only on those already published is biased toward shorter intervals. To account for this, we performed a Kaplan-Meier analysis, with “survival time” estimated as the time from preprint posting to journal publication. Although the term “preprint” implies that the manuscript will eventually be published in a scientific journal, we know from prior studies that a large proportion of preprints may never reach journal publication [1, 15]. Therefore, to account for the expected plateau in the survival curve, we used a non-mixture parametric cure model which considers that a proportion of the preprints may be “cured,” i.e., that they will never be published in a scientific journal [16]. Our results suggest that only about 20% of preprints in iSearch will eventually become journal publications; preprints in bioRxiv and medRxiv are more likely to be published (45% and 33% respectively).

During the COVID-19 pandemic, rapid access to surveillance data and scientific findings was important for developing effective responses to control disease spread and reduce morbidity and mortality. Governments of many countries developed public websites reporting data on COVID-19 cases and deaths, such as https://coronavirus.data.gov.uk/ which provides numbers of COVID-19 cases, COVID-19 related deaths, and vaccinated people for the United Kingdom. The United States also has a version of this, https://covid.cdc.gov/covid-data-tracker/#datatracker-home, that also provides information for the number of COVID-19 cases, COVID-19 related deaths, and vaccinated people in the United States. Government agencies also compiled databases with links to COVID-19 scientific publications, such as the iSearch database used in our study. The World Health Organization (WHO) has developed the WHO COVID-19 Research Database [17] as a comprehensive, multilingual source of scientific publications, compiled daily from searches of multiple bibliographic databases and other sources.

Preprints were another important source of scientific findings on COVID-19, especially early in the pandemic. The clearest advantage of preprints compared with traditional scientific publications is that they make results available sooner to the scientific community, a particularly urgent need during a global infectious disease outbreak [18]. Indeed, early in the pandemic, the number of COVID-19-related preprints kept pace with journal publications and the proportion of preprints reporting original research (89.8%) far exceeded that of published articles (21.3%), which included more commentaries (38.5%) and reviews (33.6%) [19]. We found that preprints outnumbered publications reporting study results only in January and February 2020, the first two months after the pandemic was recognized.

The fundamental tradeoff in preprint publishing balances speedy communication of scientific findings with public access to data and claims that have not been peer-reviewed. All preprints, including those that are never published in a journal, tend to remain online indefinitely with their own digital object identifiers (“doi numbers”), allowing them to be read and cited [20]. Even if they have been refuted or retracted, preprint findings may be presented to the public through media sources and continue to circulate [21, 22]. Media reporting on preprint findings became commonplace during the COVID-19 pandemic but a recent analysis found that only about half of media stories based on preprints acknowledged the uncertainty of the findings [23]. Failing to address uncertainty and lack of peer review may further the spread of misinformation. Also, not all journals, allow for a manuscript to be published as a preprint before journal submission [24]. This may restrict authors choice for journal submission as well as prevent manuscripts from being posted as preprints, nullifying the potential advantages of preprints.

As preprint publishing gains popularity among scientists, its status and uses are evolving. Preprint server rapidly disseminate and provide public access to research findings but not all users may recognize their limitations. Professional organizations of medical writers and publishers have proposed guidelines: for example, that authors should avoid using preprints as bibliographical references, preprints should clearly be distinguished from peer-reviewed articles, and preprint servers should use more intensive vetting procedures [20]. At the same time, some organizations advocating for more openness in science have called for and created avenues for more rigorous review of preprints [25]. For example, the Wellcome Trust supports Outbreak Science Rapid PREreview [26] to allow for structured review of preprints and provide quantitative scores in the setting of infectious disease outbreaks. Scientific publishers are also finding ways to streamline the process from preprint to publication; for example, PLOS, a leader in open access publishing, has announced new procedures for preprint authors (https://plos.org/open-science/preprints/).

The COVID-19 pandemic has demonstrated once again that the procedures and norms of scientific publishing are not just an academic matter: rapid sharing of reliable information across institutions and jurisdictions is crucial to the public health response. Scientific communication is among many social networks that the pandemic put to the test; it likewise deserves examination for lessons learned, to improve preparedness and protect trust in science and public health.

## Limitations

Although we examined a considerably larger number of preprints and publications during a longer time period than previous, similar studies, we still have incomplete information about preprints posted throughout the January 1, 2020, to December 31, 2021, study interval. Future publication of more of these preprints would change our estimates of the proportion of preprints that reach publication and the preprint-to-publication interval.

## Electronic supplementary material

Below is the link to the electronic supplementary material.

Supplementary Material 1

## Data Availability

The preprint used to generate the datasets analyzed during the study are available at iSearch, https://icite.od.nih.gov/covid19/search/.
