# Supplementary material for: COVID-19-Related manuscripts: lag from preprint to publication
Source: BMC Res Notes. 2022 Nov 5;15:340. doi: 10.1186/s13104-022-06231-9 (PMC9636814; doi:10.1186/s13104-022-06231-9)
Supplement: Supplementary file 1 — Supplementary Material 1 [file 13104_2022_6231_MOESM1_ESM.docx]

**Table: Data process detail**

| **Data Processes** | **Record Count** | **Note** |
| --- | --- | --- |
| **All iSearch Preprints** | | |
| Total Number of Preprints | 40439 | The data downloaded from iSearch on 01/20/2022  The date range is from 01/01/2020 to 12/31/2021 |
| The Number of iSearch preprints after removing duplicates | 39243 | 1196 duplicates removed |
| The Number of iSearch Preprints that have PubMed IDs | 8250 | Based on iSearch classification and automatic script |
| The Number of iSearch Preprints that have PubMed IDs   - peer-reviewed journal only | 7838 | 412 preprints in PubMed removed |
| The Number of iSearch Preprints that have PubMed IDs   - peer-reviewed journal only - journal publication date postdates preprint posting date (most recent version) | 7712 | 126 records that journal publication date precedes preprint posting date |
| **1^st^ Version bioRxiv and medRxiv Preprints** | | |
| The Number of 1^st^ version iSearch bioRxiv/medRxiv preprints | 20699 | The data downloaded from iSearch on 01/20/2022  The date range is from 01/01/2020 to 12/31/2021 |
| The Number of 1^st^ version iSearch bioRxiv/medRxiv preprints after removing duplicates | 20698 | 1 duplicate removed |
| The Number of iSearch bioRxiv/medRxiv that have PubMed IDs | 7511 | Based on iSearch classification and automatic script |
| The Number of iSearch bioRxiv/medRxiv that have PubMed IDs   - peer-reviewed journal only | 7402 | The records were selected from the above dataset for bioRxiv and medRxiv types only |
| The Number of iSearch bioRxiv/medRxiv that have PubMed IDs   - peer-reviewed journal only - journal publication date postdates preprint posting date (1^st^ version) | 7358 | 44 records that journal publication date precedes preprint posting date (1^st^ version) |
